# Adherence to *Plasmodium vivax *malaria treatment in the Brazilian Amazon Region

**DOI:** 10.1186/1475-2875-10-355

**Published:** 2011-12-13

**Authors:** Elza A Pereira, Edna AY Ishikawa, Cor JF Fontes

**Affiliations:** 1 Secretaria de Estado de Saúde Publica do Pará, Departamento de Controle de Endemias, Belem, (PA), Brazil; 2 Nucleo de Medicina Tropical, Universidade Federal do Pará, Belem, (PA), Brazil; 3 Nucleo de Estudos de Doenças Infecciosas e Tropicais de Mato Grosso, Universidade Federal de Mato Grosso, Rua Tulipas 316, Cond Florais Cuiaba, Cuiaba, MT, CEP: 78049-412, Brazil

**Keywords:** Malaria, Treatment, Adherence

## Abstract

**Background:**

Patients' adherence to malaria treatment is an important factor in determining the therapeutic response to anti-malarial drugs. It contributes to the patient's complete recovery and prevents the emergence of parasite resistance to anti-malarial drugs. In Brazil, the low compliance with malaria treatment probably explains the large number of *Plasmodium vivax *malaria relapses observed in the past years. The goal of this study was to estimate the proportion of patients adhering to the *P. vivax *malaria treatment with chloroquine + primaquine in the dosages recommended by the Brazilian Ministry of Health.

**Methods:**

Patients who were being treated for *P. vivax *malaria with chloroquine plus primaquine were eligible for the study. On the seventh day of taking primaquine, they were visited at their home and were interviewed. The patients were classified as probably adherent, if they reported having taken all the medication as prescribed, in the correct period of time and dosage, and had no medication tablets remaining; probably non-adherent, if they reported not having taken the medication, in the correct period of time and dosage, and did not show any remaining tablets; and certainly non-adherent, if they showed any remaining medication tablets.

**Results:**

242 of the 280 patients reported having correctly followed the prescribed instructions and represented a treatment adherence frequency (CI95%) of 86.4% (81.7%-90.1%). Of the 38 patients who did not follow the recommendations, 27 (9.6%) were still taking the medication on the day of the interview and, therefore, still had primaquine tablets left in the blister pack. These patients were then classified as certainly non-adherent to treatment. Although 11 patients did not show any tablets left, they reported incorrect use of the prescribed therapy regimen and were considered as probably non-adherent to treatment.

**Conclusions:**

Compliance with the *P. vivax *malaria treatment is a characteristic of 242/280 patients in the surveyed region. However, the group of non-adherent patients can have an impact on the magnitude of transmission and relapses of *P. vivax *infections currently observed in the studied area. Simple practices can be introduced in the healthcare services in order to improve compliance with the treatment prescribed.

## Background

In 2010, 306,908 cases of malaria were recorded in Brazil, 85% of which were caused by *Plasmodium vivax*. The majority (99.7%) of such cases occurred in the Amazon Region [1], where *P. vivax *infections are treated with a chloroquine plus primaquine regimen. Although this regimen is effective in different dosages and/or duration of treatment for *P. vivax *malaria [2], several studies have reported therapy failures including those conducted in the Brazilian Amazon region [3,4].

An unsuccessful malaria treatment can be determined by factors related to the patient, the parasite, and the treatment itself. Regarding patient-related aspects, it has been emphasized the patients' adherence to the treatment as an important factor in determining the therapeutic response to anti-malarial drugs, when a large number of treatments occur without medical supervision [5]. It contributes to the patient's complete recovery and prevents the emergence of parasite resistance to anti-malarial drugs. In Brazil, the low compliance with malaria treatment probably explains the large number of *P. vivax *malaria relapses observed in the past years. This fact is more significant in areas with an unstable population, since it becomes difficult to follow up on patients during treatment, thus confirming the observations of Duarte and Gyorkos (2003), who emphasized low adherence to treatment as an important factor in predicting subsequent episodes of malaria in the same individual [6].

Several factors are associated with low adherence to the treatment of malaria. The most emphasized factors are bad instructions or patient not understanding the treatment instructions, patient forgetting to take the medication, disintegration or loss of the medication tablets, occurrence of side effects [7] and, in some instances, use of alcohol concomitantly with the medication, which was observed mainly in camping, settlement, and mining areas (Pereira, EA personal information). In the case of drugs such as chloroquine, which induce a quick clinical improvement with few adverse effects, the disappearance of the symptoms is also cited as an important reason for the early suspension of the treatment with primaquine [8]. Inadequate prescription and dispensing of anti-malarials by health professionals are recognized as important causes of the low adherence to anti-malarial treatment [9]. Studies performed to evaluate the use of primaquine to prevent relapses of *P. vivax *malaria have considered that such information is necessary to develop strategies for the rational use and control of anti-malarial drugs [7,10].

The main goal of this study was to estimate the proportion of patients adhering to the *P. vivax *malaria treatment with chloroquine + primaquine (in the dosages recommended by the Brazilian Ministry of Health) among patients from five municipalities in the Amazon region. The secondary goal of the study was to describe the patient reports on the information they received at the community health center during medication prescription and dispensing procedures.

## Methods

### Areas of study

The study was conducted in the Amazon state of Pará, in the municipalities of Alenquer, Goianesia do Pará, Itaituba, Maraba, and Santarem, where high incidence of malaria has been reported in the past years. In addition to the high incidence of malaria, the selection of these municipalities also took into account the accessibility and infrastructure of the healthcare services that provide diagnosis and treatment of the disease at local level.

### Patients in the study

The sample size was determined using an expected low adherence proportion of 20% [6], with significance level of 95%, and precision level of 5%. Therefore, the study would require the participation of 240 patients. Since it was not possible to predict the number of patients who were out of home at the time of the visit, the sample size was increased by 20% in order to compensate for this expected loss. A total sample of 288 patients was required at baseline.

The participating patients submitted complete information on home address and met the following criteria: age 6 months old or older; living within the area where the study was conducted; received recent treatment for *P. vivax *monoinfection according to recommendations made by the Brazilian Ministry of Health, at the community health centre where they were first diagnosed; agreed to participate in the study; did not show serious signs or symptoms of severe malaria; were not pregnant, if woman; did not report any serious heart, liver, or kidney disease; had no history of allergies to the medications used in the treatment of *P. vivax *malaria; and did not live in the same residence as another patient already included in this study. All patients, including children, took tablets of chloroquine, as there is no liquid formulation of chloroquine available in Brazil.

The protocol standardized by the Amazon Network for the Surveillance of Anti-malarial Drug Resistance--Amazon Malaria Initiative was used in order to evaluate adherence to treatment, which can be summarized as an interview with the patient and verification of the number of remaining primaquine tablets on the day following the projected date for completion of treatment [11]. An interview data form was applied and filled out by two trained interviewers who were not members of the local healthcare team. These interviews gathered data on the patients' identification and medical history; their knowledge about malaria; instructions and care received at the community health centre where they were treated; compliance with the prescription received; reasons for an eventual failure to adhere to the treatment; adverse reactions to the treatment; current clinical status; and number of remaining anti-malarial untaken tablets at the visit. The checking for remaining tablets was made to both chloroquine and primaquine.

### Research procedures

Between June 2008 and July 2009 the records of patients who started treatment for *P. vivax *malaria in the previous week were monitored daily in the community health centres, and those who reached the seventh day of the medication - corresponding to the last dose of primaquine - were visited at their homes. After signing informed consent forms they were interviewed by one of the team members. For underage patients, the father or person responsible for the child had to sign the informed consent form.

After the interview and verification of the number of remaining tablets of medication, the patients were classified as: probably adherent, if the patient reported having taken all the medication as prescribed, in the correct period of time and dosage, and had no medication tablets remaining; probably non-adherent, if they reported not having taken the medication as prescribed, in the correct period of time and dosage, and did not show any remaining tablets; and certainly non-adherent, if the patient showed any remaining medication tablets.

All of the stages of this research were performed without interfering with the work routine at the community health centers, since the adherence-related variables are a direct function of the conditions of prescription and dispensing of medication. The community health centre's staff responsible for the diagnosis and treatment of malaria did not participate in the activities proposed for this research. The community healthcare agents in the area where the patients resided contributed only with information on how to locate the patients' homes.

### Ethical considerations

The protocol for the study was evaluated and approved by the Research Ethics Committee of the *Fundação de Medicina Tropical do Amazonas *[Amazon Tropical Medicine Foundation] under Opinion No. 3534/2002. Those patients who showed malaria symptoms during the interview were referred to the community health centres for medical re-evaluation. Those patients who reported non-adherence to the therapy instructions or still had medication tablets, were instructed to return to the community health centre if their symptoms recur.

### Result analysis procedures

Data were analysed in Epidata Analysis, version 2.2.1.171. Pearson's chi-square test with Yates correction was used to determine the relationship or association between categorical variables. A significance level of 95% was considered for all of the tests. For analysis of the factors associated with treatment adherence, patients classified as probably non-adherent were considered as certainly non-adherent, thus resulting in just two result groups for the study: adherent and non-adherent to treatment.

## Results

Two hundred and eighty (280) patients participated in this study, representing 97.2% of the calculated sample. They were interviewed in the municipalities of Alenquer (18.6%), Goianésia do Pará (24.3%), Itaituba (22.9%), Marabá (20.7%), and Santarém (13.5%). The patients were treated at community health centres located in rural, mining, settlement, and camping areas (62%), and sometimes in urban areas as well (Table 1). All of them were treated with chloroquine (total dosage: 25 mg/kg, divided over 3 days) and primaquine (0.5 mg/kg/day, for 7 days).

**Table 1:** Demographic and knowledge on malaria transmission characteristics of 280 patients infected with *Plasmodium vivax *and treated with chloroquine + primaquine in Brazilian Amazon

Characteristics		n	%
Municipality of residence	*Alenquer*	52	18.6

	*Goianésia do Pará*	68	24.3

	*Itaituba*	64	22.9

	*Marabá*	58	20.7

	*Santarém*	38	13.5

Local of treatment	*Hospital*	24	8.5

	*Emergence room*	15	5.4

	*Primary Care Unit*	67	23.9

	*Rural Health Care Center*	173	61.8

	*Others*	1	0.4

Age (years)	*< 1 *	4	1.4

	*1-4*	24	8.6

	*5-9*	29	10.4

	*10-14*	30	10.7

	*≥ 15 *	193	68.9

Gender	*Male*	186	66.4

	*Female*	94	33.6

Person who answered the interview	*Patient*	204	72.8

	*Responsible for the child*	76	27.2

			

Knowledge on how malaria is transmitted	*By mosquitos*	254	90.7

	*By water*	7	2.5

	*Do not know*	19	6.8

The majority of the participants were male (66.4%), with ages ranging from 6 months to 65 years, with a mean (SD) age of 24.8 (16.2) years. Patients over 15 years old accounted for 68.9% of the interviews. In general, the questions posed during the interviews were answered by the patients themselves. For 27.2% patients the questions were answered by mother or those responsible for them. When asked about the way malaria is transmitted, most of the patients stated that the disease is transmitted by a mosquito (Table 1).

Regarding the care received at the healthcare centres where they were treated for malaria, 280 patients answered that they did not receive any explanation about the disease and that they were not seen by a physician. 248 (88.6%) patients stated that they received both written and verbal instructions on the prescribed treatment, and 32 (11.4%) stated that they received just verbal instructions. However, almost all subjects reported having understood both written (99.2%) and verbal (97.9%) instructions. Regarding the compliance with the recommendations on medication dosage and schedule, 242 (86.4%) patients reported having correctly followed the instructions. On the possible adverse effects of the prescribed drugs, 278 (99.3%) patients stated that they did not receive any type of instruction from the person prescribing the medication (Table 2).

**Table 2:** Reports of the received and understood instructions by patients infected with *Plasmodium vivax *and treated with chloroquine + primaquine in Brazilian Amazon

Reports		n	%
Instructions received about dosage and schedule	*Writen and verbal*	248	88.6

	*Verbal only*	32	11.4

Information received about side effects	*Yes*	2	0.7

	*No*	278	99.3

Comprehension of the written instructions	*Yes*	246	99.2

	*No*	2	0.8

Comprehension of the verbal instructions	*Yes*	274	97.9

	*No*	6	2.1

Correct followed all the prescribed instructions	*Yes*	242	86.4

	*No*	38	13.6

Of the 38 patients who did not follow the recommendations, 27 (9.6%) were still taking the medication on the day of the interview and, therefore, still had primaquine tablets left in the blister pack. These patients were then classified as certainly non-adherent to treatment. Although 11 patients did not show any tablets left, they reported incorrect use of the prescribed therapy regimen and were considered as probably non-adherent to treatment. Therefore, 242 of the 280 patients represented a treatment adherence frequency (CI95%) of 86.4% (81.7%-90.1%) (Table 3). This adherence was 100% in Santarém and Alenquer, and varied in the other participating municipalities: Itaituba (84.4%), Goianésia do Pará (77.9%), and Marabá (77.6%). It is noteworthy that primaquine was the medication that all 27 of the aforementioned patients had left at the time of the interview and the maximum number of primaquine tablets left did not exceed four.

**Table 3:** Adherence classification of the patients infected with *Plasmodium vivax *and treated with chloroquine + primaquine in Brazilian Amazon

		n	%
Classification	*Probably adherent*	242	86.4

	*Probably adherent*	242	86.4

	*Certainly non-adherent*	27	9.7

Reason for non-adherence	*Forgetfulness*	22	81.5

	*Side effects*	4	14.8

	*Use of alcohol beverage *	1	3.7

When asked about the reason for not following the treatment as recommended, 22 (81.5%) of the 27 certainly non-adherent patients responded that they had forgotten to take the medication. The other five (18.5%) patients stated that it was due to the side effects of the medication, meaning worsening of the malaria symptoms, itching, and dyspepsia, or because they opted for consuming alcoholic beverages and were afraid to keep taking the drugs (Table 3).

The analysis of the factors associated with non-adherence to treatment showed that of the 38 certainly non-adherent (n = 27) and probably non-adherent (n = 11) patients, 31 (81.6%) were male, which represented a 4.4:1 male to female ratio. The proportion of certainly non-adherent patients was 16.7% (31/186) among males, and the probability of non-adherence among males was higher than among females (OR = 2.49; CI95%: 1.05-5.88; *p *= 0.05). Patients over 15 years old were not different in adherence proportion when compared to younger ones (OR = 1.54; CI 95%: 0.72-3.33; *p *= 0.35). Of the 32 patients who only received verbal instructions, 9 (28.1%) did not adhere to the treatment. Among the 248 patients instructed both verbally and in writing, the non-adherence rate was just 11.7%, and such difference was statistically significant (OR = 2.96; CI 95%: 1.25-7.00; *p *= 0.02) (Table 4).

**Table 4:** Factors associated to adherence to the *P.vivax *malaria treatment with chloroquine + primaquine in the Brazilian Amazon

Variables	Adherence n (%)		OR	CI 95%	p value
				
	No	Yes			
Gender					
*Male*	31 (16.7)	155 (83.3)	2.49	1.05 - 5.88	0.05

*Female*	7 (7.4)	87 (92.6)			

**Age**(years)					

*> 15*	28 (15.2)	156 (83.3)	1.54	0.72 - 3.33	0.35

*≤ 15*	10 (10.4)	86 (92.6)			

Type of instructions received about dosage and schedule					

*Verbal only*	9 (28.1)	23 (71.9)	2.96	1.25 - 7.00	0.02

*Writen +verbal*	29 (11.7)	219 (88.3)			

## Discussion

In this study, the patients' adherence to the *P. vivax *malaria treatment with chloroquine for 3 days + primaquine for 7 days were quantified in five municipalities in the Brazilian Amazon. A rate of 86.4% of adherent patients was observed, higher than that found by other research authors in Peru (62.2%) and Ecuador (65.9%), who used similar therapy regimen and method of evaluation [8,12]. In addition, the probable adherence rate was also higher than that found in Sri Lanka (73.8%) [13], Ecuador (58%) [12], and Venezuela (76.3%) [14]. Since professional workers responsible for diagnosing and treating the malaria did not participate in the study, and the patients were not aware that they would be visited at home during the treatment, it is unlikely that the presence of the research team on site would have influenced the behaviour of the participants in regard to adherence to treatment.

Studies to measure the compliance with the treatment of *P. vivax *malaria with chloroquine for 3 days and primaquine for 7 or 14 days, have been conducted in several countries in the Amazon region. However, there is more information available on the traditional therapy regimen primaquine for 14 days [15]. In general, adherence is lower for longer therapy regimens, when compared with the primaquine regimen for 7 days, thus reinforcing the premise that the longer the treatment, the shorter the adherence to such treatment [6,15].

The short duration of the therapy regimen with primaquine must be the explanation for a better adherence observed in this study. In fact, another study conducted in Mato Grosso, Brazil also found patient adherence rates of 84%, including patients with both *P. vivax *and *P. falciparum *infections [6]. Likewise, results of studies conducted in other regions also showed higher adherence levels for shorter and simpler therapy regimens [5]. Relative accessibility, free treatment, standardization of treatment regimens, and medication provided at the healthcare centre where the diagnosis was made, in conjunction with the repetitive use due to malaria relapses, are other factors which may contribute to the higher adherence rated found in Brazil. In this context, it is known that good prescription and dispensing of drugs and patient education are recognized as essential elements for a good adherence to anti-malarial treatment [7,10].

Although the sample in this study had a larger number of male patients, female patients showed significantly higher adherence to treatment. This difference has been previously observed [16]. Curiously, no association between adherence and the age of the patients was observed; although it is expected that children and elderly patients tend to be more adherent to therapy recommendations, as already observed in other studies [17].

Under adequate conditions, written therapy recommendation, associated or not with verbal instructions, is expected to improve adherence to treatment [18]. This fact was observed here, even when considering the low proportion of patients treated without adequate written treatment instructions. It is possible that the repetitive use of anti-malarial drugs due to disease relapses has reduced the need for written prescription instructions for these cases.

It is important not to neglect the 13.6% of non-adherent patients in these series and to reflect on the probable causes of non-adherence. Several contributing aspects for low adherence to anti-malarial treatment are described, among which one can highlight social and economic situation, cultural factors, low education level, and patient care conditions provided at healthcare centres. In addition, there are several other factors with both positive and negative effects on the anti-malarial treatment compliance [12]. It is estimated that the positive effects can be the result of factors such as knowledge about the disease, perception of the potential risks for worsening of the disease, trust in the medication, ease of administering the drug, shorter treatment time, and participation of healthcare agents in monitoring the treatment. On the other hand, factors such as the quick disappearance of symptoms, occurrence of adverse reactions, fluctuations in the local population numbers, easy access to self-medication, difficult access to healthcare services, and bad quality of treatment provided, can have a negative effect on the adherence to treatment.

Side effects of the drugs used in the treatment of malaria have been cited as the main reason for low adherence, mainly in cases of *P. falciparum *malaria¸ whose treatment until the 1990s was done using a combination of quinine and antibiotics [9]. This was not the case in the present study, since the side effects are not very frequent when using chloroquine + primaquine for *P. vivax *malaria, in spite of the fact that some patients stated that adverse reactions were the cause of their non-adherence to treatment. The use of chloroquine is enough to alleviate the symptoms in the first 24 hours of treatment, although a radical cure of the infection can only occur after the round of primaquine treatment is completed. The patient's lack of knowledge about the slower primaquine hypnozoitocidal effect, in conjunction with the lack of adequate prescription instructions, may result in the early suspension of the drugs and also constitute a negative influence on adherence.

Factors not assessed in this study could be speculating as the cause of the occurrence of non-adherence to treatment with primaquine. For example, it is common in the Amazon region eating wild foods such as "açai", leading to discontinuation of treatment with primaquine, because they believe that food is incompatible with primaquine. However, this popular practice is more common in Marajo Island, located in northern Pará, far from the region where the study was conducted.

A differentiated pattern of non-adherence to treatment was observed among the municipalities studied. This could have been due to the general characteristics of the population of each municipality, since three of them (Marabá, Goianésia do Pará, and Itaituba) are areas of high population fluctuation caused by several settlements, camping sites and mining sites. Under these conditions, low education level, alcoholism, and little concern with heath-related issued are common; and all such conditions are associated with low adherence to the treatment of malaria [19].

## Conclusion

The results presented herein show that although adherence to treatment is a characteristic of most patients with *P. vivax *malaria in the surveyed region, a certain percentage of such patients do not comply with therapy instructions. This group of non-adherent patients can have an impact on the magnitude and relapses of *P. vivax *infections currently observed in the studied area. Therefore, some simple practices can be introduced to in the healthcare services in order to improve compliance with the treatment prescribed. For example, a check list containing all the steps to be taken to ensure the good quality of the orientation during patient care would be a valuable instrument for the healthcare provider, so he or she would not run the risk of forgetting crucial information such as the need to fully complete the therapy regimen.

## Competing interests

The authors declare that they have no competing interests.

## Authors' contributions

EAP, EAYI and CJFF, conceived and designed the study, EAP and EAYI conducted the field work and supervised the interview data collection. CJFF and EAP participated in data management and analysis and wrote the draft of the manuscript. All authors contributed during writing, read and approved the manuscript.
